# Analysis by RP-HPLC and Purification by RP-SPE of the *C*-Tetra(*p*-hydroxyphenyl)resorcinolarene *Crown* and *Chair* Stereoisomers

**DOI:** 10.1155/2019/2051282

**Published:** 2019-04-16

**Authors:** Alver A. Castillo-Aguirre, Zuly Jenny Rivera Monroy, Mauricio Maldonado

**Affiliations:** Departamento de Química, Facultad de Ciencias, Universidad Nacional de Colombia-Sede Bogotá, Bogota 11001, Colombia

## Abstract

A method for the separation of stereoisomer mixture of the octol *C*-tetra(*p*-hydroxyphenyl)calix[4]resorcinarene that was obtained by an acid cyclocondensation reaction between resorcinol and benzaldehyde is reported in this paper. A crude product from octol formation reaction was analyzed by reverse-phase high-performance liquid chromatography (RP-HPLC), and two well-resolved signals corresponding to the *crown* and *chair* isomers were found. A reverse phase in solid-phase extraction (RP-SPE) protocol allowed the separation of the two stereoisomers with high purity of each isomer. Finally, the crude and purified stereoisomers were characterized by using FT-IR, ^1^H-NMR, and ^13^C-NMR techniques, confirming the chemical identity of the two isomers and the efficiency in the separation process.

## 1. Introduction

**Figure 1:**
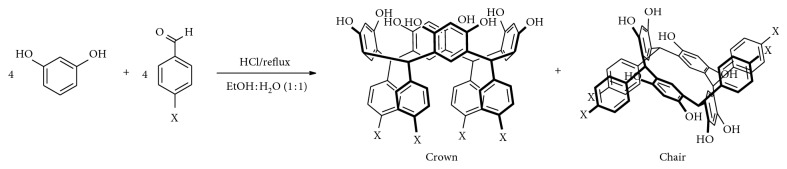
Synthesis of aromatic resorcinolarenes.

Resorcinarenes are macromolecules with four resorcinol rings linked by methylene bridges [1, 2], and they have wide applications in chemical separations [3, 4], in NMR techniques as solvating agents [5], and as chemosensors [6–9], among others. Resorcinolarenes are synthesized by the acid-catalyzed cyclocondensation of resorcinol with aliphatic or aromatic aldehydes [10–12]. Acid-catalyzed condensation reaction by choosing aromatic aldehyde as a starting material usually produces a conformational mixture of two stereoisomers, in different proportions, known as *chair* and *crown* (see Figure 1) [13–16]. Of these isomers, the *crown* stereoisomer is the thermodynamically stable compound; alternatively, the structure of resorcinarenes can be rigidified into a *crown* by linking the hydroxyl groups of the upper rim, which provides a higher degree of preorganization. Resorcinarenes with substituted methylene bridge are found in *crown* structure in the crystal state [17–19], and in solution, the isomer may adopt *crown* and *chair* structures [20, 21]; however, the *chair* structure can interconvert in the *crown* structure at high-temperature conditions in the presence of highly polluting metal catalysts [22, 23].

The many possible structural variations or resorcinarenes fixed in *crown* conformation and substituted with aromatic ring in the lower rim lead to potential applications as chemical receptors for molecules and ions [17, 24–27], in absorption of heavy metal ions [28], in lithographic processes [29], and as photoresistant materials for glasses [30] and are used for modification of polymeric surfaces with potential applications as stationary phase in chromatographic methods [31]. Despite the importance of this type of molecules, in terms of their applications, there is only one method reported for their separation, which includes successive crystallizations [32]. Continuing with our studies related to structure and applications of resorcinarenes [33–36], in the present investigation, we show an efficient method for the analysis of this kind of stereoisomers by RP-HPLC and it is shown that the mixture can be separated by means of the RP-SPE technique with good yields.

## 2. Materials and Methods

### 2.1. General Method

IR spectra were recorded on a Thermo Fisher Scientific Nicolet iS10 FT-IR spectrometer with a Monolithic Diamond ATR accessory and absorption in cm^−1^. ^1^H- and ^13^C-NMR spectra were recorded at 400 MHz on a Bruker Avance 400 instrument. Chemical shifts are reported in ppm, by using the solvent residual signal. The elemental analysis for carbon and hydrogen was carried out using a Thermo Flash 2000 Elemental Analyzer.

### 2.2. Synthesis of *C*-Tetra(*p*-hydroxyphenyl)resorcinarene (Stereoisomers Mixture)

We followed the method reported by Castillo-Aguirre et al. [36]. A 1,3-dihydroxybenzene solution (25 mmol) and *p*-hydroxybenzaldehyde (25 mmol) in ethyl alcohol : water (1 : 1) (50 mL) was added drop by drop to hydrochloric acid (5 mL) and was heated at reflux with constant stirring for 4 h. The crude reaction was cooled in ice bath, and the precipitate formed was filtered and washed with mixture ethyl alcohol : water (1 : 1) and so with water to remove traces of acid. The filtrate was dried under reduced pressure and was characterized by means of FT-IR, ^1^H-NMR, and ^13^C-NMR.

Violet clear solid at a yield of 91%. Mp > 250°C decomposition. FT-IR (KBr/cm^−1^): 3384 (O-H), 1172 (C-O); ^1^H-NMR, DMSO-*d*_6_, *δ* (ppm): 5.43 and 5.52 (s, 4H, ArCH), 5.92–6.10 (s, 4H, ArH, *ortho* to OH), 6.28–6.65 (m, 20H, ArH), 8.37–8.45 (s, 8OH, ArOH), and 8.68 and 8.85 (s, 4OH, ArOH).

### 2.3. Separation of the Mixture by RP-HPLC

RP-HPLC analyses were performed over a Kromasil® EternityXT 5-C18 column (4.6 × 50 mm) using an Agilent 1200 Liquid Chromatograph (Agilent, Omaha, NE, USA). A gradient ranging from 5 to 50% of solvent B (TFA 0.05% in acetonitrile) in solvent A (TFA 0.05% in water) was used. The gradient time was 8 minutes, detection was performed at 210 nm, and the flow rate was 2 mL/min. Sample concentration of *C*-tetra(*p*-hydroxyphenyl)resorcinolarene (conformational mixture) was 1.0 mg/mL, and 10 *μ*L was injected.

### 2.4. Separation of the Mixture by SPE

Supelclean™ ENVI™-18 SPE cartridges (bed wt. 5 g, volume 20 mL) were used. The cartridges were firstly conditioned by MeOH and MeCN and then equilibrated with water (containing TFA 0.05%). 100 mg of *C*-tetra(*p*-hydroxyphenyl)resorcinolarene (conformational mixture) dissolved in 600 *μ*L of DMSO was loaded on the sorbent material. After loading the mixture, the analytes were eluted with different solutions containing solvent B in solvent A, at different ratios ranging from 0 to 100% v/v.

*C*-tetra(*p*-hydroxyphenyl)resorcinarene (*crown*): violet clear solid in yield 52%. M.p. > 250°C decomposition. FT-IR (KBr/cm^−1^): 3384 (O-H), 1076 (C-O); ^1^H-NMR, *δ* (ppm): 5.52 (s, 4H, ArCH), 6.08 (s, 4H, ArH, *ortho* to OH), 6.48 (d, 8H, ArH, *J* = 8 Hz), 6.50 (s, 4H, ArH, *meta* to OH), 6.64 (d, 8H, ArH, *J* = 8 Hz), 8.45 (s, 8OH, ArOH resorcinol), 8.85 (4OH, *p*-OHAr). ^13^C-NMR, *δ* (ppm): 40.6 (ArCH), 102.1 (resorcinol C-2), 114.1 (resorcinol C-5), 121.0 (hydroxyphenyl C-3), 129.6 (hydroxyphenyl C-2), 136.0 (hydroxyphenyl C-4), 152.2 (resorcinol C-4), 152.3 (hydroxyphenyl C-1), 154.5 (resorcinol C-1).

*C*-tetra(*p*-hydroxyphenyl)resorcinarene (*chair*): cream solid in yield 48%. M.p. > 250°C decomposition. FT-IR (KBr/cm^−1^): 3401 (O-H), 1077 (C-O); ^1^H-NMR, *δ* (ppm): 5.39 (s, 4H, ArCH), 5.88 (s, 2H, ArH, *ortho* to OH), 6.06 (s, 2H, ArH, *ortho* to OH), 6.24 (s, 2H, ArH, *meta* to OH), 6.27 (s, 2H, ArH, *meta* to OH), 6.28 (d, 8H, ArH, *J* = 8 Hz), 6.38 (d, 8H, ArH, *J* = 8 Hz), 8.31 (s, 4OH, ArOH resorcinol), 8.35 (s, 4OH, ArOH resorcinol), 8.61 (s, 4 OH, *p*-OHAr). ^13^C-NMR, *δ* (ppm): 41.2 (ArCH), 113.9 (resorcinol C-2), 120.9 (resorcinol C-5), 121.9 (hydroxyphenyl C-3), 129.8 (hydroxyphenyl C-2), 134.5 (hydroxyphenyl C-4), 152.4 (resorcinol C-4), 152.6 (hydroxyphenyl C-1), 154.3 (resorcinol C-1).

## 3. Results and Discussion

**Figure 2:**
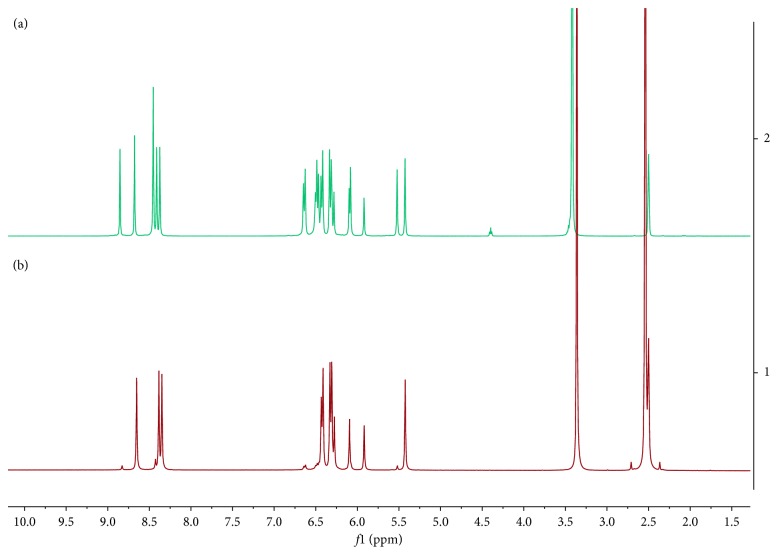
^1^H-NMR spectra to 400 MHz, DMSO-*d*_*6*_, and 293 K. (a) Conformational mixture. (b) *Chair* isomer.

**Figure 3:**
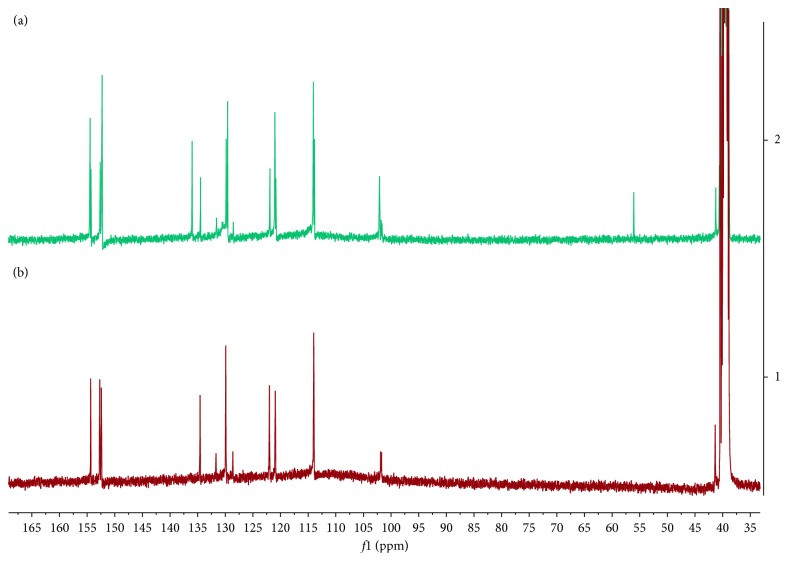
^13^C-NMR spectra to 400 MHz, DMSO-*d*_*6*_, and 293 K. (a) Conformational mixture. (b) *Chair* conformer.

As it is shown in Figure 1, the synthesis of resorcinarene was done through the acid-catalyzed cyclocondensation of 1,3-dihydrohybenzene with *p*-hydroxybenzaldehyde in a 1 : 1 mixture of ethyl alcohol and water and was heated at reflux by 4 h. The precipitate was characterized using spectral techniques, including FT-IR, ^1^H-NMR, and ^13^C-NMR (see Materials and Methods). *C*-tetra(p-hydroxyphenyl)resorcinarene had been previously synthesized [36], and the spectroscopic data agreed with those reported in this paper. In this way, the FT-IR spectrum for crude product is in agreement with the organic functionalities present in the structure of the two conformers, as it reveals the hydroxyl group stretches at 3384 cm^−1^ (O-H) and 1172 cm^−1^ (C-O), whereas the bands of the aryl substituent and the resorcinol ring are also observed. The ^1^H-NMR spectrum showed resonance signals for the aromatic hydrogen atoms for conformer mixture at 5.92 to 6.65 ppm, the methylene bridges fragments at 5.43 and 5.52, and the hydroxyl moieties (*δ* = 8.37 to 8.85 ppm) (see Figure 2(a)). In the ^13^C-NMR spectra of the product, there are two signals in the aliphatic region for carbons of the methylene bridge fragment between the aromatic rings. In the aromatic region, the carbon signals for the tetrasubstituted resorcinol unit and aryl chains also showed an increase in the number of signals. The results allow confirming the presence of two stereoisomers for resorcinarene (see Figure 3(a)).

**Figure 4:**
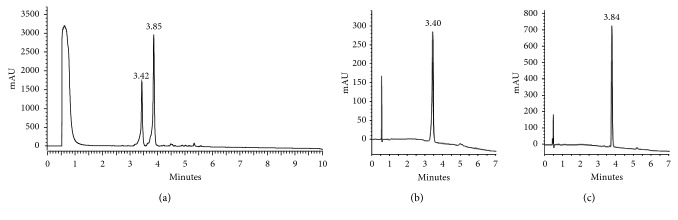
RP-HPLC profiles. (a) Conformational mixture. (b) *Crown* conformer. (c) *Chair* conformer.

After identifying the presence of the two stereoisomers in the reaction product, it was decided to analyze the mixture by means of RP-HPLC; the crude product was dissolved in DMSO, and it was analyzed using the following gradient program: 5/5/50/100/100/5/5%B at 0/1/9/9.5/11/11.5/15 minutes. The chromatographic profile showed the presence of two well-resolved signals (*R*_s_ = 2.0) at *t*_R_ 3.42 and 3.85 minutes (see Figure 4(a)). This result shows us the power of reverse phase for the separation of this kind of stereoisomer mixture.

Then we explore the possibility of scaling the separation up; we had considered RP-SPE as an attractive methodology for the purification of the stereoisomers since the results of Kamysz et al. [37] demonstrated that by means of SPE, it is possible to purify challenging molecules as antimicrobial peptides in a fast and one-step procedure, and this method furnishes products of >95–97% purity; moreover, there is no need for sophisticated equipment and consumption of mobile phase is minimal. For the preparative separation, we used commercial cartridges of 5 g and we loaded them with 100 mg of the mixture dissolved in DMSO; this solvent was removed washing the cartridge with water and then solutions, containing different growing concentrations of solvent B, were passed through. Collected fractions were analyzed by RP-HPLC, and it was found that *crown* and *chair* isomers were successfully separated (see Figures 4(b) and 4(c)) in a preparative manner.

To establish the efficiency of the separation of the two stereoisomers, the isolated products by SPE were initially analyzed by ^1^H-NMR (Figure 2(b), Table 1). The ^1^H-NMR spectra of the first product showed three different hydroxyl moieties at 8.61 ppm assigned to hydroxyl groups in the lower rim and two signals at 8.31 and 8.35 ppm corresponding to hydroxyl groups for two classes of hydroxyl groups attached to resorcinol residues in the macrocyclic system. In the aromatic region, in addition to the signals of the hydroxyphenyl substituent at 6.28 and 6.38 ppm, four signals are evidenced in the resorcinol residues, two corresponding to the protons in the *ortho* position to the hydroxyl group at 5.88 and 6.06 ppm and the other two signals corresponding to the protons in *meta* position to the hydroxyl groups at 6.24 and 6.27 ppm. In this way, these patterns were consistent with the structure of the expected *chair* stereoisomer.

On the contrary, the ^1^H-NMR spectra of the second product showed two single peaks at 8.85 and 8.45 ppm corresponding to two classes of hydroxyl groups, the first signal corresponding to a hydroxyl group in the lower rim and the second signal for the hydroxyl group in the upper rim. Additionally, all the patterns were consistent with the structure of the expected *crown* stereoisomer which has few signals in the spectrum.

As shown in Figure 3, in the ^13^C-NMR spectrum, the number of signals for the raw product contrasts with the number of signals of the pure isomer. To confirm that the two products obtained corresponded to the cyclic stereoisomers, the ^13^C-NMR was also obtained. The ^13^C-NMR spectra of *chair* stereoisomer (see Figure 3(b)) displayed characteristic signals of hydroxyphenyl substituent (121.9, 129.8, 134.5, and 152.6 ppm), and the aromatic carbons of resorcinol appeared at 113.9, 120.9, 152.7, and 154.3 ppm. The signal at 41.2 ppm confirmed the presence of a methylene bridge fragment between the aromatic rings. In the same way, the *crown* stereoisomer showed nine signals, one at 40.6 ppm confirmed the presence of a methylene bridge fragment between the aromatic rings. The aromatic region showed four signals for hydroxyphenyl substituent at 121.0, 129.6, 136.0, and 152.3 ppm and for the carbons of resorcinol residue appeared at 102.1, 114.1, 152.2, and 154.5 ppm (see Table 1).

Finally, comparing conventional crystallization and SPE technique for the separation of stereoisomers of *C*-tetra(*p*-hydroxyphenyl)calix[4]resorcinarene, we found great advantages. Specifically, in a typical crystallization procedure, a small amount of crude product of *C*-tetra(*p*-hydroxyphenyl)calix[4]resorcinarene was dissolved in acetone and stirred for 1 h; after cooling to 0°C, the remaining residue was filtered and the mother liquor was dried. Acetone extracts most of the *chair* stereoisomer and the residue left with a mixture of *chair* and *crown* stereoisomers, the process must be repeated several times to achieve high purity, and the yield of process is very low for the *crown* stereoisomer although the stereoisomer *chair* is obtained with good yield. In contrast with SPE technique, the separation can be carried out with less solvent which reduces purification times and enhances yields in comparison with conventional crystallization. On the contrary, previously we used as separation procedure classic column chromatography and AcOEt-benzene 8 : 2 as eluent solvent [36], and this experimental protocol showed similar results to those obtained by the crystallization technique. The proposed method using SPE has been successfully applied to the separation of the mixture of *C*-tetra(*p*-hydroxyphenyl)calix[4]resorcinarene and it has advantages in terms of selectivity, time of separation, yield, and reproducibility.

## 4. Conclusions

In this study, a mixture of stereoisomers of *C*-tetra(*p*-hydroxyphenyl)calix[4] resorcinarene was obtained from conventional synthesis, and the crude was analyzed by RP-HPLC. These stereoisomers were successfully separated by RP-SPE technique. The ^13^C and ^1^H-NMR spectra of each separated conformer confirmed that the separation method was efficient. The developed method was applied and has the advantages of high sensitivity, low running cost, and simple operation and could be applied in the analysis of analogue systems of mixtures of stereoisomers.

## Figures and Tables

**Table 1:** Chemical shifts for pure stereoisomers *crown* and *chair* in ^1^H-NMR and ^13^C-NMR spectra.

General structure	Proton or carbon	*δ* (ppm)			
		^1^H-NMR		^13^C-NMR	
		*Crown*	*Chair*	*Crown*	*Chair*
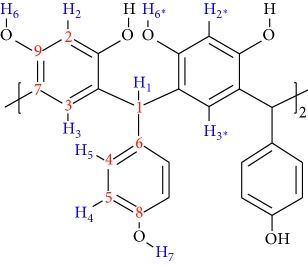	1	5.52	5.39	40.6	41.2
	2	6.08	5.88	102.1	113.9
	2^*∗*^	—	6.06	—	—
	3	6.50	6.24	114.1	120.9
	3^*∗*^	—	6.27	—	—
	4	6.48	6.28	121.0	121.9
	5	6.64	6.38	129.6	129.8
	6	8.45	8.31	136.0	134.5
	6^*∗*^	—	8.35	—	—
	7	8.85	8.61	152.2	152.4
	8	—	—	152.3	152.6
	9	—	—	154.5	154.3

^∗^Resolved signal corresponding to the protons of the chair conformer.

## Data Availability

The IR and NMR data used to support the findings of this study are included within the article.
